# Characterization of Large-Scale Mitochondrial DNA Rearrangements Using Complementary Short- and Long-Read Sequencing Approaches

**DOI:** 10.3390/genes17101161

**Published:** 2026-09-22

**Authors:** Jing Wang, Joe Chan, Mian Umair Ahsan, Amy Goldstein, Rebecca D. Ganetzky, Sonal Sharma, James T. Peterson, Douglas C. Wallace, Tanaya Jadhav, Ramakrishnan Rajagopalan, Kai Wang

**Affiliations:** 1 Division of Genomic Diagnostics, The Children’s Hospital of Philadelphia, Philadelphia, PA 19104, USA; 2 Department of Pathology and Laboratory Medicine, Perelman School of Medicine, University of Pennsylvania, Philadelphia, PA 19104, USA; 3 Raymond G. Perelman Center for Cellular and Molecular Therapeutics, The Children’s Hospital of Philadelphia, Philadelphia, PA 19104, USA; 4 Mitochondrial Medicine Frontier Program, Division of Human Genetics, Department of Pediatrics, The Children’s Hospital of Philadelphia, Philadelphia, PA 19104, USA; 5 Department of Pediatrics, Perelman School of Medicine, University of Pennsylvania, Philadelphia, PA 19104, USA; 6 Center for Mitochondrial and Epigenomic Medicine, Division of Human Genetics, The Children’s Hospital of Philadelphia, Philadelphia, PA 19104, USA

**Keywords:** mitochondrial DNA (mtDNA), mtDNA deletions and duplications, PacBio long-read sequencing, Oxford Nanopore Technologies long-read sequencing, Illumina short-read sequencing

## Abstract

Background: Large-scale mitochondrial DNA (mtDNA) rearrangement, including deletion or duplication, is one of the important causes of primary mitochondrial disease. In clinical practice, short-read sequencing (SRS)-based assays typically detect these rearrangements by identifying regional mtDNA copy-number changes but generally do not directly distinguish deletions from duplications. As a result, mtDNA duplications may be misclassified as deletions, potentially leading to inaccurate molecular diagnoses and genotype–phenotype correlations. This study aimed to evaluate the utility of long-read sequencing (LRS) for the definitive characterization of large-scale mtDNA rearrangements. Methods: Two individuals who had been reported to possess single large-scale mtDNA deletions using SRS-based clinical tests were re-evaluated because their clinical presentations were not consistent with typical mtDNA deletion syndromes. Existing SRS data were reanalyzed, and the suspected rearrangements were independently characterized using both Pacific Biosciences (PacBio) and Oxford Nanopore Technologies long-read sequencing platforms. Results: Reanalysis of SRS data suggested that the reported deletions were more consistent with mtDNA duplications. This finding was confirmed by both PacBio and Oxford Nanopore LRS in one case. Reclassifying the deletion to a duplication in this case substantially improved genotype–phenotype interpretation and better explained the atypical clinical features observed in this individual. However, LRS could not capture sufficient long reads that give direct evidence of duplication in the second case due to low heteroplasmy of the mtDNA structural variant. Therefore, this case remains inconclusive. Conclusions: SRS and LRS provide complementary information for the evaluation of large-scale mtDNA rearrangements. SRS can sensitively identify mtDNA large deletions, but it cannot distinguish mtDNA deletion from duplication. On the other hand, LRS can provide direct structural information and confirm duplication. However, LRS may have limited sensitivity for low-level heteroplasmic structural variants. An integrated approach incorporating both methods, together with clinical correlation and reanalysis of potentially discordant findings, may improve the characterization and interpretation of mtDNA rearrangements.

## 1. Introduction

Mitochondrial DNA (mtDNA) is essential for cellular energy production, encoding key components of the oxidative phosphorylation (OXPHOS) system. Large-scale mtDNA rearrangements, including deletions and duplications in kilobase sizes, have been reported [1,2]. Single large-scale mtDNA deletions (SLSMDs) typically lead to overlapping clinical features, including syndromes such as Pearson syndrome (PS), Kearns–Sayre syndrome (KSS), the KSS spectrum, and chronic progressive external ophthalmoplegia (CPEO) and CPEO plus [3]. In contrast, large-scale mtDNA duplications have been reported in patients with KSS and myopathy phenotypes [1,2], but they are less well characterized. The original identification of mtDNA duplication was through Southern blot and restriction fragment length polymorphism (RFLP) analysis. Many of the reported duplications resulted from the merging of wild-type mtDNA and a deletion molecule [4,5]. In reported cases, the deletion sizes are usually greater than 7 kilobases (Kb) [4,5]. Studies have demonstrated that cybrids with 100% mtDNA duplication exhibited no respiratory chain or protein synthesis defects compared to wild-type cells. In contrast, cell lines with 100% mtDNA deletions exhibited a complete impairment of respiratory chain function and oxidative phosphorylation [1]. These results suggested that simple SLSMD and mtDNA duplication may be associated with different phenotypes or severities for primary mitochondrial diseases.

Accurate detection and differentiation between mtDNA deletions and duplications are critical for understanding disease mechanisms, prognosis, and potential therapeutic approaches. Common methods for identifying mtDNA structural variants include long-range PCR, Southern blotting, and short-read next-generation sequencing (SRS). While long-range PCR and Southern blotting remain traditional diagnostic tools, current mtDNA tests are mostly SRS-based, providing higher sensitivity and accuracy for mtDNA deletion detection. However, standard analysis procedures cannot detect duplications or distinguish deletions from duplications [6].

In this study, we applied PacBio HiFi and Oxford Nanopore long-read sequencing (LRS) for structural variant analysis [7] in two patients previously reported to have SLSMDs through standard clinical tests, but lacking a clinical presentation consistent with an SLSMD syndrome diagnosis. LRS confirmed mtDNA duplications in one case, while the other case remained inconclusive due to the low heteroplasmy level of the mtDNA structural variant.

## 2. Materials and Methods

### 2.1. Clinical Presentations of Patients

Patients in this study were evaluated by clinicians at the Mitochondrial Medicine Frontier Program (MMFP) at the Children’s Hospital of Philadelphia (CHOP). This study was performed under Institutional Review Board (IRB) approved protocols (#23-020790, Wang, PI).

Case 1: An African-American 16-year-old male presents with short stature, failure to thrive, developmental delay, dysarthria, migraines, optic atrophy, no ptosis or ophthalmoplegia, bilateral sensorineural hearing loss, bilateral hip dysplasia, scoliosis, and mitral valve prolapse with left ventricular hypertrophy, with a pacemaker implanted. Brain imaging revealed basal ganglia calcifications. Metabolic laboratory testing revealed elevated serum levels of GDF15, lactate, and alanine, as well as an elevated ratio of beta-hydroxybutyrate to acetoacetate. He reported severe fatigue and was found to have hemolytic anemia requiring mitral valvuloplasty. Immediately following surgery, he was found to have lower extremity hemiplegia, and brain imaging was repeated. At that time, he was found to have a watershed injury in addition to metabolic strokes in multiple locations, including cortical/subcortical regions, the corpus callosum, and the midbrain, as well as putaminal necrosis; however, it did not reveal classic Kearns–Sayre syndrome (KSS) findings, such as white matter changes or globus pallidus lesions. Due to these atypical features, the diagnosis of KSS was questioned, and further genetic studies were undertaken at this time (see below). Two years later, he presented with altered mental status, and imaging had progressed significantly, with confluent rarefaction and cavitation of white matter, extensive symmetric lesions of the brainstem, and dentate nuclei. He developed progressive respiratory failure and passed away in the hospital.

Case 2: A 36-year-old female presents with exercise intolerance, fatigue, unilateral ptosis, and moderate to severe sensorineural hearing loss on the right side. Additionally, she has a history of Ig A nephropathy with resultant end-stage renal disease requiring renal transplant, metabolic syndrome with obesity and prediabetes, peripheral neuropathy, imbalance, depression, anxiety, and gross motor developmental delay. Metabolic laboratory testing revealed mildly elevated serum alanine and C5DC-carnitine levels. Serum GDF15 (1987 pg/mL, ref < 750) was also elevated. Her brain MRI was normal.

Both patients had undergone exome tests, which were non-diagnostic. However, mtDNA analysis by SRS revealed the presence of SLSMDs in both patients. Since neither patient exhibited the classic presentation of SLSMD syndromes, further analysis of mtDNA was conducted using PacBio and Oxford Nanopore long-read sequencing.

### 2.2. Short-Read Next-Generation Sequencing (SRS)

Total DNA, including nuclear and mitochondrial DNA, was extracted using a commercially available DNA isolation kit (Gentra Systems Inc., Minneapolis, MN, USA). Full-length mitochondrial DNA was amplified using long-range PCR (LR-PCR) and sequenced using paired-end short reads on an NovaSeq 6000 (Illumina, Inc. San Diego, CA USA). Reads were aligned to NC_012920.1 using Novoalign. Deletions were called using Pindel and filtered to retain only deletions over 200 bp. SNVs were called using the Freebayes Variant caller [6].

Deletion heteroplasmy from SRS: when large-scale mtDNA deletions were detected by SRS-based clinical testing, a follow-up ddPCR assay on a Bio-Rad QX200 Droplet Digital PCR System using a ddPCR Supermix for Probes (No dUTP) kit (#1863024, Bio-Rad, Hercules, CA, USA) was performed to quantify the deletion heteroplasmy. In brief, two ddPCR reactions were carried out, one targeting the mtDNA commonly deleted region (MTND4) and the second targeting a region that is rarely involved in large mtDNA deletions (MT-RNR2). These two ddPCR reactions were used to determine the number of non-deleted mtDNA molecules and total mtDNA molecules, respectively. The deletion heteroplasmy level was calculated as [1 − (ND4/RNR2)] × 100% [6].

### 2.3. Long-Read Sequencing (LRS) by PacBio HiFi

Total DNA, including nuclear and mitochondrial DNA, was extracted using a commercially available DNA isolation kit (PUREGENE DNA Isolation Kit, Qiagen, MD, USA). A total of 4500 ng of DNA was diluted to 30 ng/μL and sheared using either a Covaris G-tube (Covaris, LLC. Woburn, MA, USA) or the Megaruptor 3 shearing kit (Diagenode, LLC. Denville, NJ, USA). Sheared samples were prepared for sequencing using the SMRTbell Library Prep Kit 3.0 and Sequel lle 3.2 binding kit, then sequenced on a PacBio Sequel lle system according to the manufacturer’s instructions (PacBio Inc., Menlo Park, CA, USA). Each sample was sequenced using one SMRTcell on a PacBio Sequel lle system without any amplification, and the HiFi reads were aligned to the reference genome GRCh38 using pbmm2 v1.13.1 with the iso-seq preset, which allows for lower gap extension penalties.

### 2.4. Long-Read Sequencing by Oxford Nanopore—Targeted PCR

Mitochondrial DNA was amplified using New England Biolabs’ LongAmp Hot Start *Taq* 2X Master Mix (NEB #M0533S). Each 50 μL reaction contained 40 ng of genomic DNA, 25 μL of 2X master mix, 2 μL of 10 μM forward primer, 2 μL of 10 μM reverse primer, 1.5 μL of DMSO, and nuclease-free water to volume. The forward primer sequence was 5′-CCGCACAAGAGTGCTACTCTCCTC-3′, and the reverse primer sequence was 5′-GATATTGATTTCACGGAGGATGGTG-3′. Thermocycling conditions consisted of an initial denaturation at 94 °C for 30 s, followed by 30 cycles of 94 °C for 15 s, 56 °C for 30 s, and 65 °C for 16 min, with a final extension at 65 °C for 10 min. These conditions were designed to amplify the 16–20 kb mitochondrial genome. PCR products were purified using AMPure XP beads (Beckman Coulter #A6388) at a 0.4X bead ratio, washed twice with 80% ethanol, and eluted in 25 μL of nuclease-free water.

Approximately 1 μg (~100 fmol) of purified PCR product was used for ONT library preparation using the Nanopore Ligation Sequencing Kit V14 (ONT SQK-LSK114) (Oxford Nanopore, Oxford, UK) according to the manufacturer’s instructions. The library was sequenced on a Flongle flow cell for 24 h. The raw POD5 data were basecalled using Dorado v0.9.0 with the dna_r10.4.1_e8.2_400bps_sup@v5.0.0 model. The resulting reads were aligned to the GRCh38 human mitochondrial reference sequence.

### 2.5. Long-Read Sequencing by Oxford Nanopore—PvuII Linearization

To linearize full-length mitochondrial DNA and mtDNA harboring duplications, 1 μg of genomic DNA was combined with 5 μL of 10X NEBuffer and 1 μL of PvuII-HF (NEB #R3151S) in a total volume of 50 μL with nuclease-free water. The reaction was incubated at 37 °C for 1 h. DNA was purified using AMPure XP beads (Beckman Coulter #A6388) at a 0.4X bead ratio, washed twice with 80% ethanol, and eluted in 50 μL of nuclease-free water.

The entire PvuII-digested DNA sample was used for ONT library preparation using the Nanopore Ligation Sequencing Kit V14 (ONT SQK-LSK114) (Oxford Nanopore, Oxford, UK) according to the manufacturer’s instructions. The library was sequenced on a MinION flow cell for 72 h. The raw POD5 data were basecalled using Dorado v1.0.2 with the dna_r10.4.1_e8.2_400bps_sup@v5.2.0 model. The resulting reads were aligned to the GRCh38 human mitochondrial reference sequence.

### 2.6. Computational Analysis of Long-Read Data to Assess Mitochondrial Duplication Structure

To evaluate whether candidate mitochondrial structural variants were consistent with tandem duplication, we used a breakpoint-aware reference reconstruction strategy followed by read-level alignment filtering. Because the mitochondrial genome is circular, we first re-linearized the mitochondrial sequence by cutting the circular reference at a position outside the candidate duplicated interval. This placed the candidate rearranged region and its flanking sequence in a linear coordinate system suitable for evaluating reads that span the predicted duplication junctions.

For each candidate event, we generated a duplication-aware mitochondrial reference in which the suspected duplicated sequence was represented twice in tandem. Conceptually, the duplicated interval was defined by two sequence blocks flanking the origin in the original reference sequence, denoted here as E and S. In the duplication-aware reference, these blocks occur as two copies: E1-S1 for the first copy and E2-S2 for the second copy. Thus, the modeled tandem-duplication reference contains the structure:

**left-flanking sequence − E1 − S1 − E2 − S2 − right-flanking sequence**

where E1 and E2 represent the same reference-derived sequence block, and S1 and S2 represent the second reference-derived sequence block repeated in the duplication model. This construction allows the expected duplication-specific structure to be represented explicitly as a single linear reference.

Long reads were aligned to the duplication-aware mitochondrial reference using minimap2 with the map-pb (PacBio reads) or map-ont (Nanopore reads) preset and default parameters. The resulting BAM files were processed with a custom python script. Unmapped and secondary alignments were excluded, and reads below a minimum length threshold were removed. In the final analysis, read support was assessed at the level of individual alignment records rather than by merging split alignments from the same read. Therefore, a read was considered informative only if a single primary alignment record spanned all required diagnostic regions on the duplication-aware reference.

To identify reads supporting the duplication model, we defined diagnostic intervals around the key junctions created by the tandem-duplication reference. These intervals were chosen relative to the E1-S1/E2-S2 structure so that support required continuous read alignment across both duplicated copies. Specifically, intervals were placed near the transition into the first duplicated copy, the internal duplication junction between S1 and E2, and the distal end of the second duplicated copy. Equivalently, a supporting read had to align across the reference positions, distinguishing the first copy from the second copy, including the sequence near E1-S1, the duplicated-copy junction S1-E2, and the sequence near E2-S2. This requirement ensured that the read was not merely aligning to one copy of the repeated sequence, but instead spanned enough of the modeled duplication reference to distinguish a tandem duplication from a deletion-like or ambiguous alignment pattern.

For each retained alignment record, aligned reference blocks were extracted from the CIGAR string. A read was classified as duplication-supporting only if the blocks from a single alignment record overlapped all diagnostic intervals. Candidate supporting reads were then filtered to remove alignments containing very large insertion or deletion operations, since such CIGAR patterns can artificially bridge distant regions and mimic structural-variant-spanning alignments. For each alignment, the largest insertion or deletion operation in the CIGAR string was calculated, and alignments exceeding a predefined maximum indel threshold were excluded.

The final set of candidate duplication-supporting reads consisted of sufficiently long, primary alignment records that overlapped all duplication-diagnostic intervals on the duplication-aware mitochondrial reference and did not contain large CIGAR-defined indels. Read names from these alignments were exported for downstream inspection and visualization. This approach specifically required a single continuous alignment to span the modeled duplicated copies, providing read-level evidence for tandem duplication rather than relying on split-read aggregation or ambiguous coverage of only one duplicated segment.

As an additional, less stringent read-selection strategy, we also considered a secondary set of diagnostic intervals designed to identify shorter candidate reads that may support the duplication model but do not span both duplicated copies and the outer flanking sequence in a single alignment. In this approach, candidate reads were required to overlap the internal duplication-specific junction between S1 and E2, together with internal regions of both E1 and E2 located at least 100 bp away from the adjacent E1/S1 and S1/E2 junctions. This criterion was intended to cast a wider net for reads containing evidence of the duplicated structure while avoiding intervals immediately adjacent to the junction boundaries. However, because this relaxed pattern does not require full traversal of both duplicated copies and their flanks, some reads arising from deletion-like alleles or ambiguously rearranged molecules could also satisfy the same overlap criteria. Using this secondary criterion, we identified a candidate duplication/deletion-informative read in sample 22U; because the alignment pattern alone could not unambiguously distinguish duplication from deletion under this relaxed definition, we performed follow-up PCR experiments to resolve the underlying structural configuration.

## 3. Results

### 3.1. mtDNA Analysis of Case 1

A large mtDNA deletion, m.3577_15546del11970, was detected in the blood sample using the capture-based SRS method by Blueprint Genetics. The heteroplasmy in the blood sample was reported to be 60.5%. However, the mitochondrial medicine clinicians who followed up with the patient did not think that Case 1’s clinical presentation was consistent with KSS. A follow-up mtDNA analysis in our laboratory detected the exact same deletion in a urine sample from Case 1 using long-range PCR (LR-PCR) and SRS (Figure 1A,B). Digital droplet PCR (ddPCR) analysis determined that the deletion heteroplasmy was 43% in the urine sample. In addition, a novel de novo homoplasmic missense variant, m.5983G>A (p.Gly27Glu), in the *MT-COI* gene was detected in both the urine and blood samples. This variant was not detected in the maternal saliva sample. Notably, this variant has not been reported in the scientific literature, variant databases such as MITOMAP (http://www.mitomap.org, accessed on 15 July 2026), or population studies such as gnomAD (the Genome Aggregation Database). It occurred at a highly conserved amino acid residue. Computational prediction tools such as APOGEE [8] suggest that this variant may affect protein function. This variant is classified as a variant of uncertain significance, leaving it unclear how it contributes to Case 1’s clinical presentation.

Case 1 has been included in a study on mtDNA pathogenic variants in the haplogroup L population, which indicated that the phenotype is optic atrophy and ophthalmoplegia [9]. Despite the large size of the deletion (over 10 Kb) and the high heteroplasmy in both blood and urine samples, the patient did not exhibit the typical mtDNA deletion syndrome presentations. Therefore, further analysis of the mtDNA structural variant was initiated using PacBio LRS. The LRS results revealed that the undeleted part of the mtDNA, marked as sections S and E (Figure 1C), was actually in tandem duplication. The full length of mtDNA with this duplication spans over 20 Kb (Figure 1D). This duplication was present in all reads that captured the breakpoints. These findings are illustrated in Figure 1E using a read alignment plot generated by Ribbon [10]. The structure of the duplication is illustrated in Figure 2.

Collectively, the results suggest that instead of calling the mtDNA structural change as a deletion of m.3577_15546 (Figure 2B), it is actually the result of the concatenation of a full-length mtDNA molecule and a deletion molecule, leading to a duplication of m.15546_3577dup4600 (Figure 2C). Notably, there is an 11 bp microhomology (ccccctcccca) at the breakpoints.

### 3.2. mtDNA Analysis of Case 2

The previous mtDNA test on a buccal sample from this individual reported a deletion at m.79_13637del13559 with a heteroplasmy of less than 15% by GeneDx. The follow-up mtDNA analysis on a muscle sample from this case confirmed the same breakpoints using long-range PCR followed by SRS (Figure 3A). The presence of the deletion was confirmed by a long-range PCR gel image (Figure 3B). However, upon reviewing the SNPs across the mitochondrial genome using the long-range PCR primer set P6, it was observed that SNPs within the m.13637_79 region were predominantly present at approximately 50% heteroplasmy, while those outside of this region were mostly homoplasmic, as expected (Figure 3C, Supplementary Table S1). These findings were confirmed using a separate long-range PCR primer set (P8) and SRS (Figure 3C,D). Notably, both P6 and P8 primer sets are located within the m.13637_79 region (Figure 3D). To further investigate, we applied a third long-range PCR primer set (P7), located outside the m.13637_79 region, for long-range PCR and SRS. No heteroplasmic SNVs were detected in the P7 results, as expected. Collectively, these findings suggest the presence of an additional mtDNA fragment corresponding to the m.13637_79 region in this individual’s mitochondrial genome. Haplogroup analysis revealed that the heteroplasmic SNPs present solely in the P6 and P8 results belonged to haplogroup J1c7, while the patient’s haplogroup was identified as N1b1b. These findings suggested that the extra mtDNA fragment in the m.13637_79 range could be a duplication of m.13637_79dup3011, rather than a deletion as previously reported. The heteroplasmy of this duplication was quantified as 7% using ddPCR. Interestingly, this mtDNA structure change was not detected in the mother’s buccal sample.

However, the PacBio and ONT LRS results were inconclusive. Both methods showed a single read supporting duplication (Figure 4B,C). The PacBio LRS results show only one sequencing read encompassing the duplicated sections A and E (Figure 4B). We were unable to capture the long sequencing reads that encompass the entire mitochondrial genome plus the tandem duplication. The breakpoints were identical to those previously detected by SRS. The mtDNA structural change is illustrated in Figure 5. The ONT LRS results also show only one read that encompasses the duplicated section (Figure 4C). This read does span the entire mitochondrial genome; however, there is an unexpected 150-base insertion. Since PCR and ONT long-read sequencing are inherently biased toward shorter DNA fragments, further analysis was performed to confirm whether this read represented a true duplication or a PCR or sequencing artifact. To this end, mtDNA was linearized using the PvuII restriction enzyme, which cuts at m.2652 and is present only in the wild-type and tandem-duplication haplogroups. Subsequent analysis of the linearized data showed no evidence of a duplication, with only the wild-type haplogroup detected (Figure 4D). Based on the collective results, it is inconclusive whether the tandem duplication m.13637_79dup3011 (Figure 5C) exists.

## 4. Discussion

The clinical significance of large-scale mtDNA structural changes, such as SLSMDs, and their association with primary mitochondrial diseases has been well established [3,11,12]. However, the exact mechanisms underlying the formation of large-scale mtDNA deletions and duplications remain unclear. Several mechanisms have been proposed, including replication slippage, copy-choice recombination, and impaired double-strand break (DSB) repair [13,14,15,16]. Further studies are needed to validate these hypotheses and clarify how mtDNA damage, secondary structures, and microhomology sequences at deletion breakpoints contribute to deletion formation.

The MITOMAP and MitoBreak databases documented hundreds of unique single deletions [17,18]. However, reports of mtDNA duplications are limited [2,4,19], with most cases identified using Southern blot, LR-PCR, or restriction fragment length polymorphism (RFLP) analysis. These conventional methods have been phased out due to being highly labor-intensive, low-throughput, and insensitive. SRS has been widely used in mtDNA analysis in both research and clinical settings. Duplicated mtDNA consisting of a full-length mtDNA molecule and a deleted mtDNA molecule has been reported [1]. Unlike the diploid nuclear genome, cells contain hundreds to thousands of mtDNA copies. SRS-based copy number analysis relies on detecting relative copy number gains or losses across different regions (Figure 1A and Figure 3A). Unfortunately, there is no specific bioinformatics pipeline that can definitively distinguish large-scale mtDNA deletions and duplications, as the breakpoint sequences at deletion and duplication junctions are identical [20]. As a result, mtDNA duplications are often misclassified as deletions.

The duplication observed in Case 1 falls into this category. In such duplications, direct repeats or microhomologies at the boundaries are often present [1], similar to those seen at common mtDNA deletion breakpoints [17]. This suggests that these types of deletions and duplications may arise through a shared mechanism or that one could be derived from the other [1]. A previous study demonstrated that transmitochondrial cell lines harboring mtDNA duplications exhibited no significant defects in oxygen consumption or protein translation levels compared to wild-type cells. In contrast, cell lines with mtDNA deletions showed severe deficiencies in respiratory chain activity and mitochondrial protein translation. In general, patients with a predominantly duplicating mtDNA structural variant may exhibit milder or atypical phenotypes [1]. These findings suggest that duplicated mtDNAs, especially those composed of concatenated full-length and deleted molecules, may not have a significant physiological impact, as seen in SLSMD. LRS analysis of Case 1 revealed that nearly all abnormal mtDNA molecules were duplications, with no observed deletions. This could explain the absence of typical SLSMD-related symptoms despite the high reported heteroplasmy level. A similar mtDNA duplication case was recently reported in two siblings and the mother, all affected with dilated cardiomyopathy [21]. However, the clinical presentations in these reported cases are not consistent with what we observed in Case 1. It is important to note that these types of duplications may be unstable and prone to recombination, potentially resulting in mtDNA deletions and SLSMD presentations in some patients [2]. Since the exome test on Case 1 did not identify any causal variants in the nuclear genome, the collective evidence suggests that the novel de novo missense variant m.5983G>A (p.Gly27Glu) in the *MT-COI* gene may contribute to, or potentially account for a significant portion of, the patient’s severe neurological manifestations. On the other hand, the mtDNA duplication may represent a distinct structural finding with a less direct impact on the phenotype. Functional studies will be necessary to determine the pathogenicity and clinical significance of this novel *MT-COI* variant.

Case 2 has an mtDNA structural variant that requires further investigation. While the breakpoint analysis from SRS supports an SLSMD of m.79_13637del13559 in this case (Figure 3A and Figure 5B), there are no similar SLSMDs reported in the MITOMAP [17] and MitoBreak [18] databases. More importantly, if the deletion is genuine, it would remove over 13.5 kilobases of the mitochondrial genome, including over half of the D-loop region, which spans base positions m.16024_576. The D-loop is a crucial structure involved in the regulation of mtDNA replication and transcription [22]. The remaining 3 kilobases of the mtDNA fragment, with only a fraction of the D-loop, would be challenging to replicate, keep intact, and detect in multiple tissue types, including buccal swabs and muscle. In addition, the presence of numerous heteroplasmic SNVs in the non-deleted region m.13637_79, which belong to a different haplogroup (Supplementary Table S1), strongly suggests the presence of additional mtDNA material corresponding to the m.13637_79 region. Unfortunately, due to the low heteroplasmy level of this structural variant, the LRS carried out by PacBio and ONT could not capture a sufficient number of long reads that could provide direct evidence to support a tandem duplication (Figure 4). While it is unlikely that this mtDNA fragment originated from the case’s native mtDNA, as it belongs to a different haplogroup, its exact origin remains unknown. A BLAT search of the human nuclear genome did not reveal any sequences with high homology to this fragment (Supplementary Figure S1), and it has not been reported in studies on mega-NUMTs [23]. The long-range PCR SRS is designed to avoid NUMT interference [24]. Furthermore, the structural variant was confirmed by two separate sets of long-range PCR primers, making it even less likely that the additional mtDNA fragment originated from a NUMT.

This structural variant was not detected in the mother’s buccal sample. Several studies have reported the presence of numerous high-heteroplasmy SNVs in probands, ultimately revealing paternal mtDNA transmission [24,25,26]. However, without an available paternal sample for Case 2, we cannot rule out the possibility that the fragment was inherited from the father. Notably, in previously documented cases of biparental mtDNA inheritance, heteroplasmic variants were observed throughout the entire mitochondrial genome, resembling an admixture of two mitochondrial genomes from distinct haplogroups [24,25]. In contrast, Case 2 exhibited an admixture that only impacted a 3011 bp mtDNA fragment. While the exact mechanism underlying this structural change remains unknown, it is likely distinct from the mechanisms described in cases of full mtDNA duplication [24,25].

Because mtDNA is present in hundreds to thousands of copies per cell, SRS-based structural variant analysis commonly relies on regional differences in sequencing depth to identify copy-number gains or losses. A particular limitation is that the breakpoint sequence generated by a deletion and that generated by a tandem duplication can be identical when the duplicated molecule consists of a full-length mtDNA molecule and a corresponding deleted mtDNA molecule. Consequently, SRS-based approaches may identify the presence of an abnormal mtDNA population but cannot always distinguish whether the underlying structure represents a deletion or a duplication. While SRS is well suited for detecting and screening for mtDNA sequence and copy-number abnormalities, LRS can provide direct structural information when informative molecules are sufficiently represented. Neither approach alone necessarily determines the complete molecular architecture of every mtDNA structural variant.

This study has several limitations. First, the number of cases is small, with only two cases and confirmed mtDNA duplication in only one case. Second, the low heteroplasmy of the structural variant in Case 2 limited the number of informative long reads generated by both PacBio and ONT sequencing, preventing definitive resolution of its molecular architecture. Third, heteroplasmy quantification by ddPCR should be interpreted with caution, particularly for low-level heteroplasmy such as that observed in Case 2. Thus, the ddPCR values should be considered approximate estimates of variant abundance rather than definitive measurements of structural-variant heteroplasmy [27].

In summary, these two cases illustrate the complexity of characterizing large-scale mtDNA structural variants using current SRS sequencing approaches. Case 1 demonstrates that an mtDNA rearrangement interpreted as a deletion by SRS may represent a predominantly duplicative structure when evaluated using LRS, whereas Case 2 highlights that even LRS may be insufficient to resolve a low-level and potentially complex mtDNA rearrangement. These findings underscore the complementary nature of SRS and LRS and the importance of integrating sequencing results with orthogonal methods and clinical findings. Given the small number of cases and the unresolved molecular architecture of Case 2, these observations should be considered illustrative rather than generalizable. Larger studies incorporating diverse mtDNA structural variants and additional orthogonal approaches will be needed to better define their molecular mechanisms and clinical significance.

## Figures and Tables

**Figure 1:**
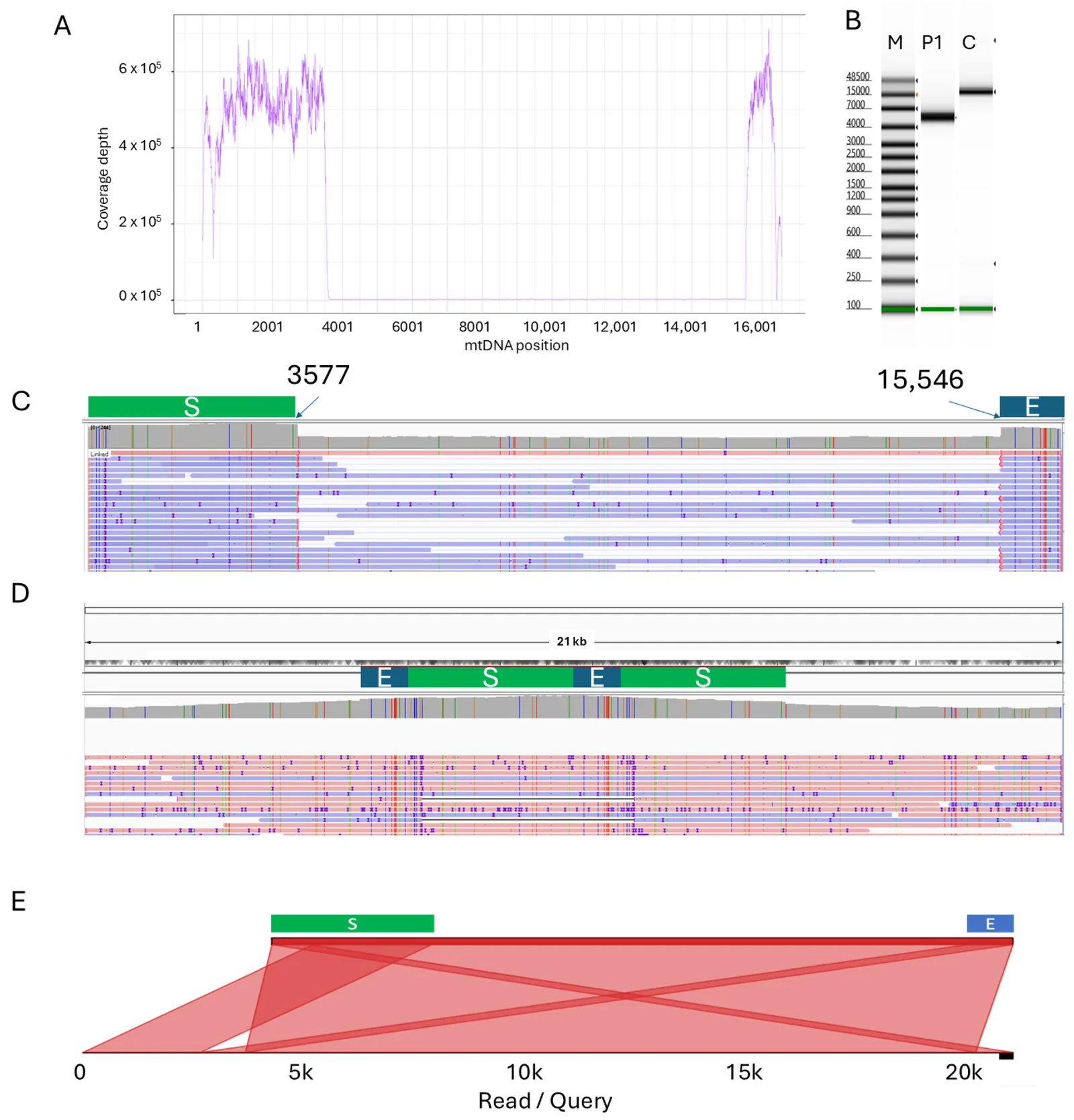
mtDNA structural variant detected in Case 1. (**A**) Coverage data from short-read sequencing indicated a large-scale mtDNA deletion. X-axis: coverage depth from long-range PCR-based SRS; Y-axis: mtDNA position. (**B**) A long-range PCR gel image showing a deletion in this sample. M: molecular marker, P1: Case 1 patient, C: deletion-negative sample. The amplification band represents the full-length mtDNA in lane C and the deleted molecule in lane P1. (**C**,**D**) PacBio LRS data aligned to the human mitochondrial genome. C is the normal genome and shows increased coverage of a putatively deleted/duplicated region. D shows the same data aligned to a mitochondrial genome with the expected tandem duplication added. (**E**) The Ribbon plot illustrates a representative read from LRS that exhibits evidence of duplication. The top line represents the full-length mtDNA reference, while the bottom line corresponds to a single LRS read exceeding 20 kilobases. The shaded areas indicate the mapped reads from LRS to the reference sequences. Notably, both the S and E segments were found to be duplicated in this long read from LRS.

**Figure 2:**
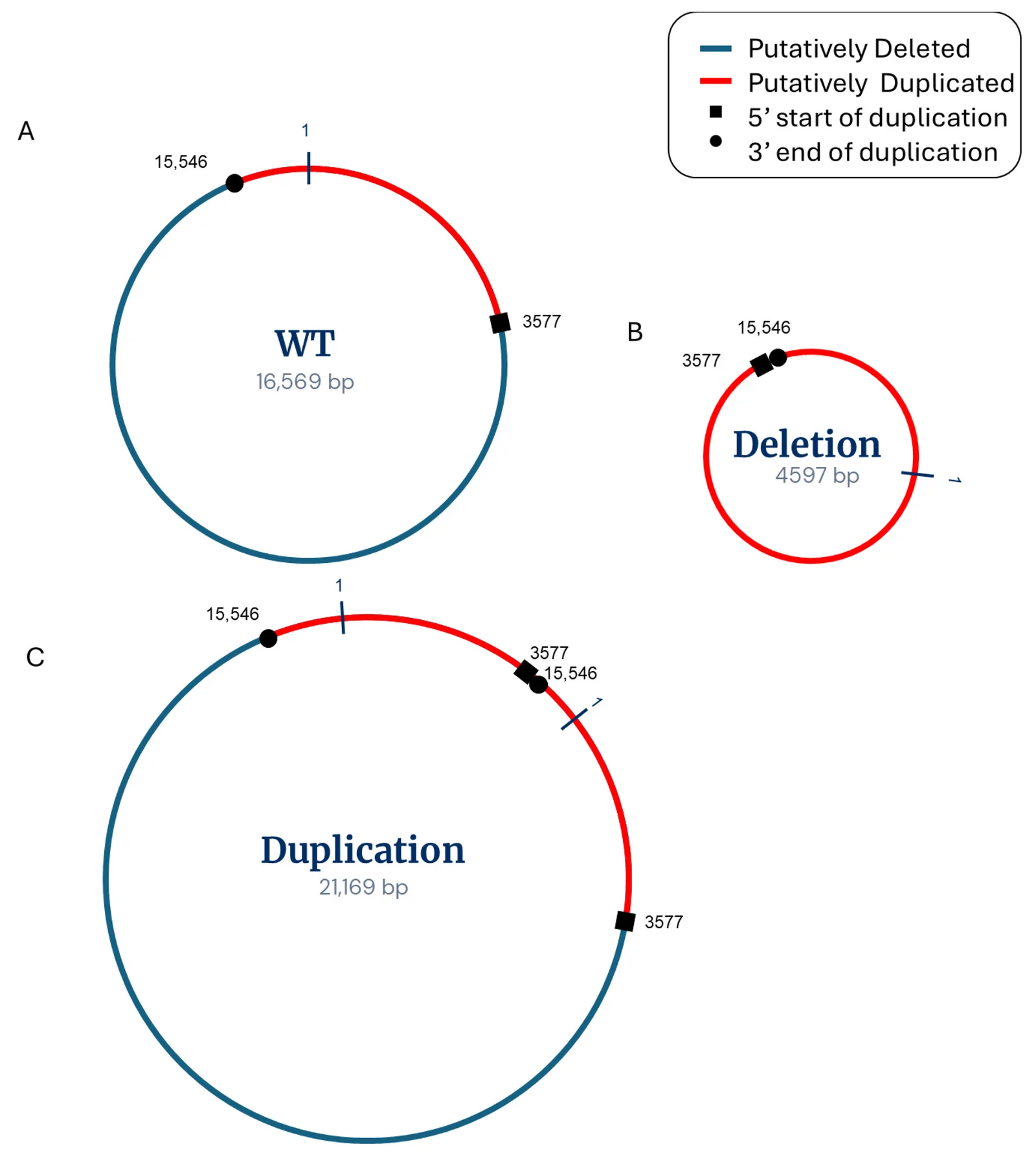
Possible structures of Case 1’s mitochondria, with the wild-type (**A**), deletion (**B**), and tandem duplication (**C**).

**Figure 3:**
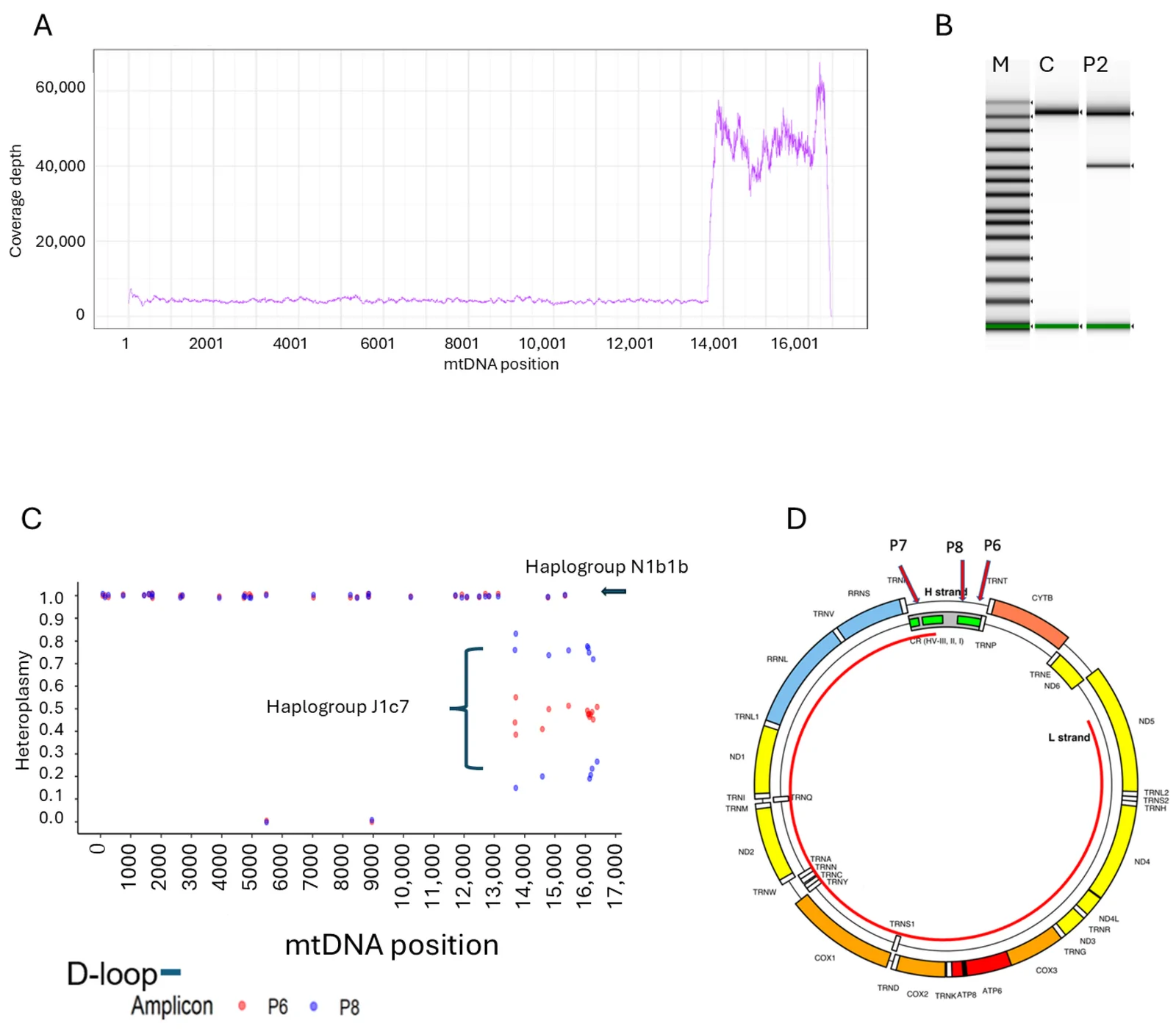
mtDNA structural variant detected in Case 2. (**A**) Coverage data from short-read sequencing indicated a large-scale mtDNA deletion. X-axis: coverage depth from long-range PCR-based SRS; Y-axis: mtDNA position. (**B**) A long-range PCR gel image showing a deletion in this case. M: molecular marker, C: deletion negative, P2: Case 2 patient. The amplification band represents the full-length mtDNA in lane C. Lane P2 contains both the full-length and deleted molecules. (**C**) Large numbers of heteroplasmic variants are clustered in the m.13637_79 region. The red dots represent heteroplasmic variants detected by long-range PCR primer set P6 at approximately 50% heteroplasmy. The purple dots indicate the same set of SNVs detected by long-range primer set P8 at either 75% or 25% heteroplasmy. The difference in heteroplasmy levels between P6 and P8 is likely due to variations in amplification efficiencies between wild-type and mutant mtDNA molecules in the two different PCR reactions. This set of heteroplasmic variants belongs to haplogroup J1c7, while the patient’s mtDNA belongs to haplogroup N1b1b. The two bars at the bottom indicate the D-loop region. (**D**) Location of the three long-range PCR primers with respect to the mitochondrial genome. The P6 and P8 primer sets are situated in the m.13637_79 region, while the P7 primer is located outside this region. The reverse and forward primers are positioned back-to-back consecutively (reverse ←•→ forward) to amplify the entire length of the circular mtDNA.

**Figure 4:**
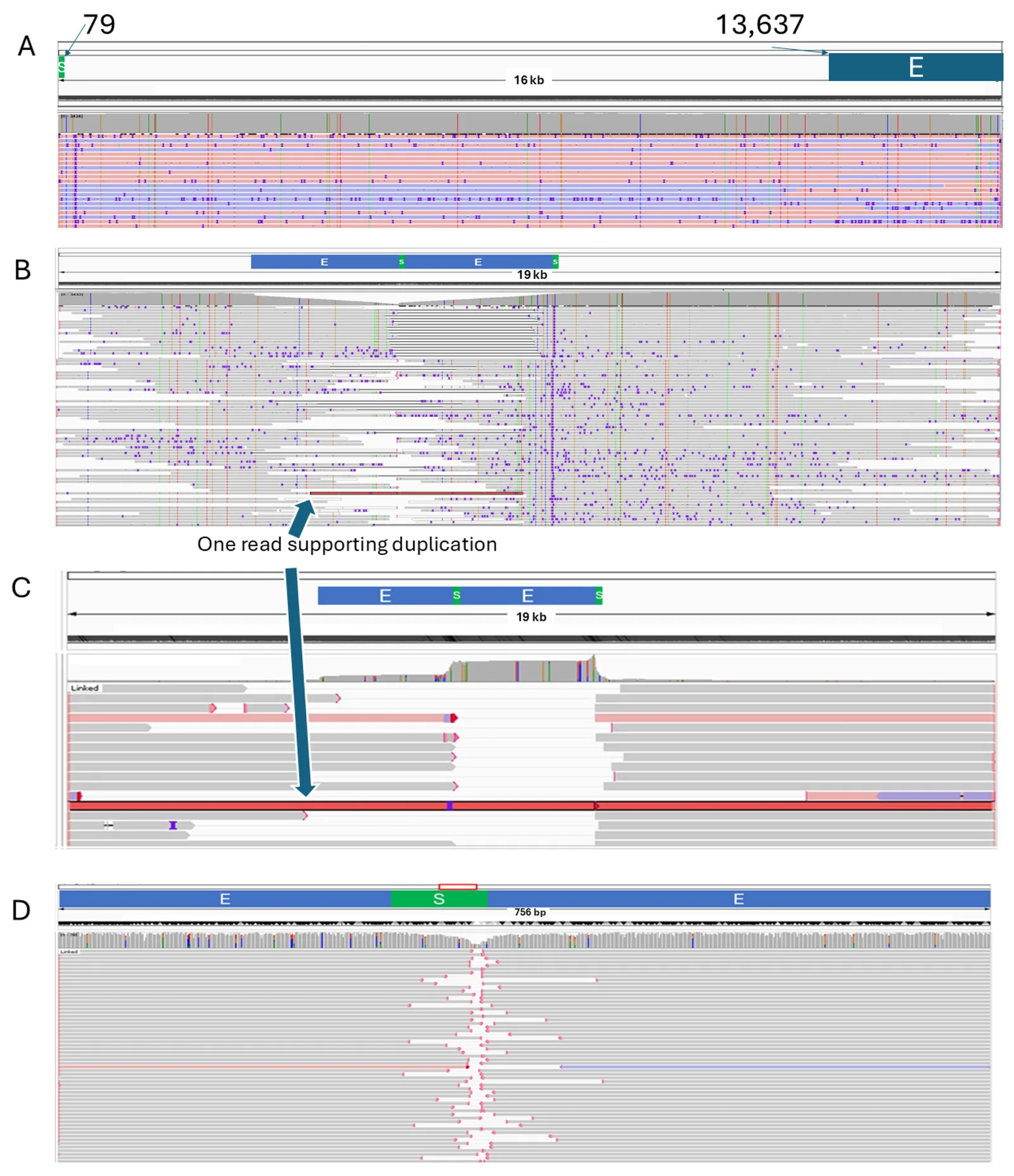
Various IGV plots of the Case 2 reads aligned to the mitochondrial genome. (**A**) The PacBio LRS read. (**B**,**C**) The one read “support” for a tandem duplication, with B being the PacBio data and C being the ONT data. (**D**) PvuII-digested data showing no reads that span the tandem duplication.

**Figure 5:**
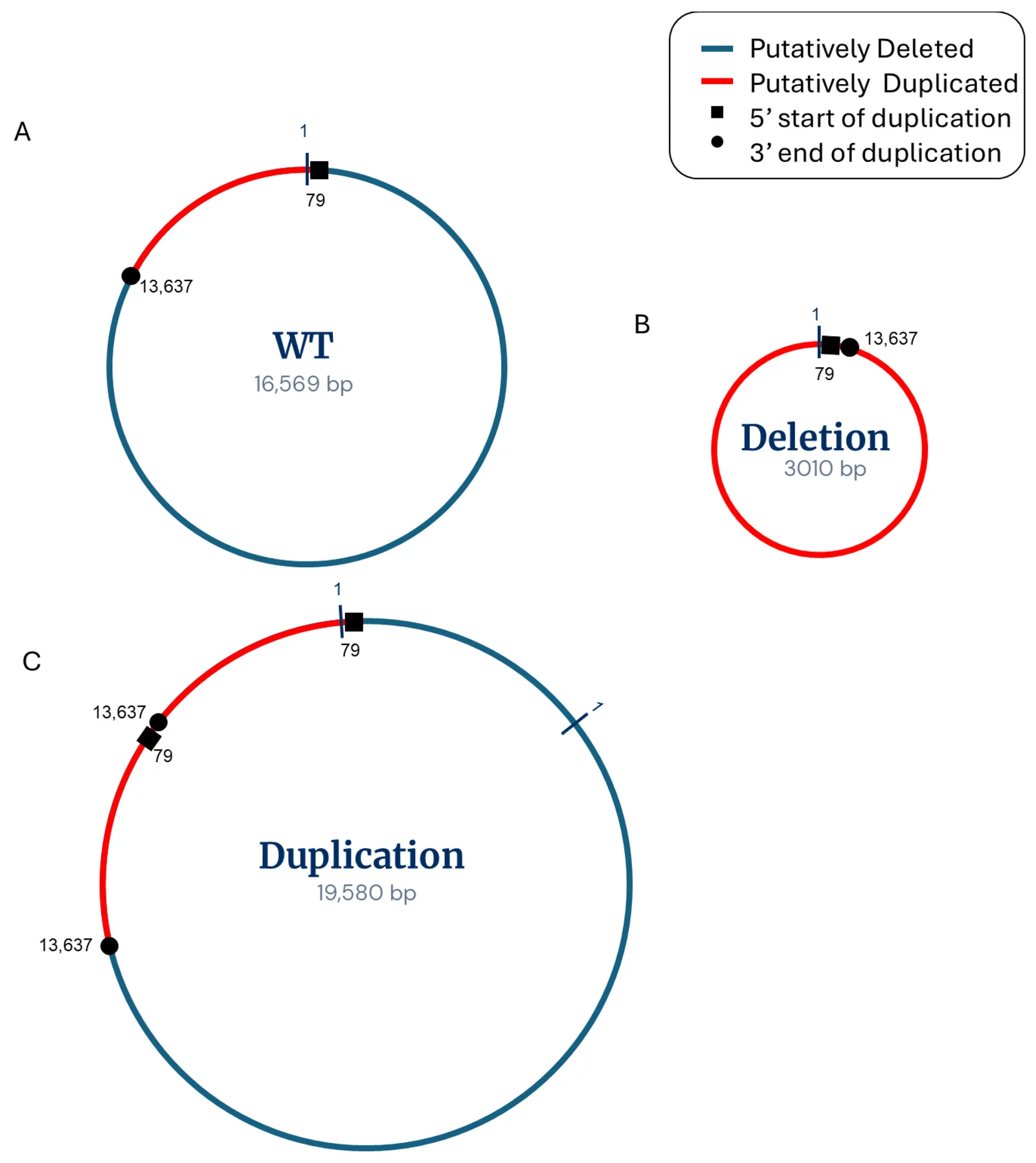
Possible structures of Case 2’s mitochondria, with wild-type (**A**), deletion (**B**), and tandem duplication (**C**).

## Data Availability

The original contributions presented in this study are included in the article/Supplementary Materials. Further inquiries can be directed to the corresponding author.

## Supplementary Materials

The following supporting information can be downloaded at: https://www.mdpi.com/article/10.3390/genes17101161/s1, Figure S1: The extra mtDNA fragment is unlikely from NUMTs. The back-to-back position of forward and reverse long-range PCR primers makes it only suitable for circular mtDNA and unlikely to amplify NUMTs in the nuclear genome. Furthermore, the mtDNA m.13637_97 fragment does not have a good match in the nuclear genome; Table S1: The mtDNA variants detected in Case 2 through short-read sequencing using long-range primers P6, P7, and P8. VAF presents heteroplasmy level of each variant.
