# Supplementary material for: Characterization of Large-Scale Mitochondrial DNA Rearrangements Using Complementary Short- and Long-Read Sequencing Approaches
Source: Genes (Basel). 2026 Sep 22;17(10):1161. doi: 10.3390/genes17101161 (PMC13704522; doi:10.3390/genes17101161)
Supplement: Supplementary file 1 [file genes-17-01161-s001.zip › genes-4505111-supplementary.pdf]

Figure S1: The extra mtDNA fragment is unlikely from NUMTs. The back-to-back position of forward and reverse long-range PCR primers makes it only suitable for circular mtDNA and unlikely to amplify NUMTs in the nuclear genome. Furthermore, the mtDNA m.13637_97 fragment does not have a good match in the nuclear genome.

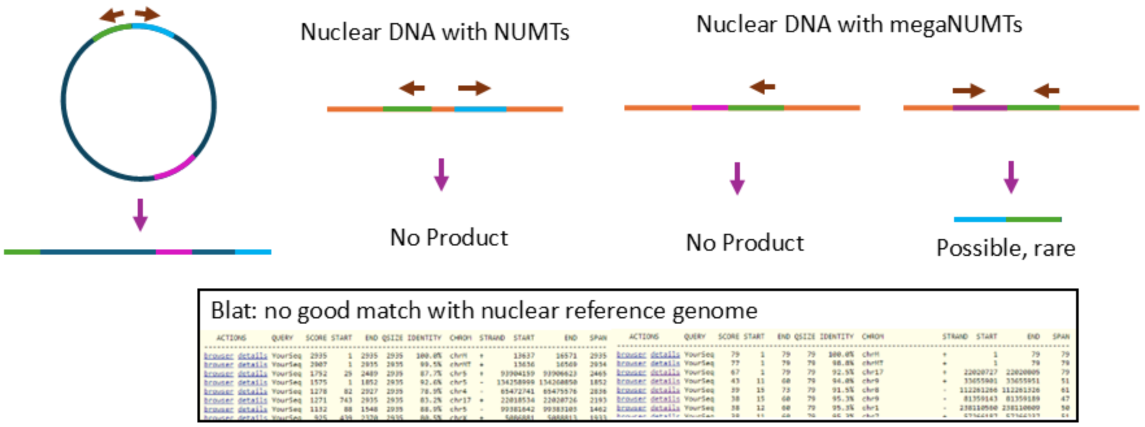

Table S1. The mtDNA variants detected in Case 2 through short-read sequencing using long-range primers P6, P7, and P8. VAF presents heteroplasmy level of each variant.

**mtDNA variant**	**VAF P6**	**VAF P8**	**VAF P7**
m.73A>G	1.0	1.0	0.999
m.152T>C	0.999	0.999	1.0
m.189A>G	0.014	0.015	0.011
m.207G>A	0.042	0.037	0.054
m.263A>G	1.0	0.999	0.0
m.750A>G	1.0	1.0	1.0
m.1438A>G	1.0	1.0	1.0
m.1598G>A	0.999	1.0	1.0
m.1703C>T	1.0	0.997	1.0
m.1719G>A	1.0	1.0	1.0
m.2639C>T	0.999	0.999	1.0
m.2706A>G	1.0	1.0	0.999
m.3921C>A	0.999	0.999	1.0
m.4735C>A	0.999	0.998	0.999

m.4769A>G	1.0	1.0	1.0
m.4917A>G	0.999	0.999	1.0
m.4960C>T	0.999	0.999	0.999
m.5471G>A	0.999	0.999	1.0
m.5480A>G	0.003	0.003	0.005
m.7028C>T	1.0	0.999	0.999
m.8251G>A	1.0	0.999	0.999
m.8472C>T	1.0	0.999	1.0
m.8836A>G	0.999	0.999	1.0
m.8860A>G	1.0	1.0	1.0
m.8959G>A	0.008	0.005	0.008
m.10238T>C	1.0	1.0	1.0
m.11719G>A	1.0	0.999	1.0
m.11928A>G	1.0	0.999	1.0
m.12092C>T	1.0	0.999	1.0
m.12501G>A	1.0	1.0	1.0
m.12705C>T	1.0	1.0	1.0
m.12822A>G	0.999	0.999	1.0
m.13129C>T	0.999	0.997	1.0
m.13681A>G	0.437	0.763	0.0
m.13708G>A	0.556	0.823	0.0
m.13710A>G	0.394	0.147	1.0
m.14581T>C	0.418	0.205	1.0
m.14766C>T	1.0	1.0	1.0
m.14798T>C	0.503	0.744	0.0
m.15326A>G	1.0	1.0	1.0
m.15452C>A	0.506	0.765	0.0
m.16069C>T	0.493	0.779	0.0
m.16092T>C	0.477	0.764	0.0
m.16126T>C	0.474	0.751	0.0
m.16145G>A	0.455	0.195	1.0
m.16176C>A	0.472	0.205	1.0
m.16223C>T	0.476	0.23	0.999
m.16261C>T	0.445	0.715	0.0
m.16390G>A	0.509	0.266	0.999
m.16519T>C	0.425		1.0
